# Spirituality, moral injury and mental health among Chinese health professionals

**DOI:** 10.1192/bjo.2021.972

**Published:** 2021-07-19

**Authors:** Zhizhong Wang, Faten Al Zaben, Harold G. Koenig, Yuanlin Ding

**Affiliations:** Department of Epidemiology and Statistic, School of Public Health at Guangdong Medical University, Dongguan, Guangdong, China; Division of Psychiatry, Department of Medicine, King Abdulaziz University, Jeddah, Saudi Arabia; Division of Psychiatry, Department of Medicine, King Abdulaziz University, Jeddah, Saudi Arabia, and Departments of Psychiatry and Medicine, Duke University Medical Center, Durham, NC, USA; Department of Epidemiology and Statistic, School of Public Health at Guangdong Medical University, Dongguan 560001, Guangdong, China

**Keywords:** Moral injury, depression, healthcare professional, mediation, COVID-19, spirituality

## Abstract

**Background:**

Moral injury has been found to be prevalent among healthcare professionals during the COVID-19 public health crisis.

**Aims:**

The present study examines the relationship between spirituality, moral injury, and mental health among physicians and nurses in mainland China during the COVID-19 pandemic.

**Method:**

An online cross-sectional study was conducted involving 3006 physicians and nurses in mainland China, where the COVID-19 pandemic has caused high rates of hospital admission and death. The Moral Injury Symptoms Scale-Health Professional was administered, along with measures of mental health and spirituality. Hierarchical linear regression modelling was used to examine the mediating and moderating role of moral injury in the relationship between spirituality and mental health.

**Results:**

Spirituality was positively correlated with moral injury (β = 2.41, *P* < 0.01), depressive symptoms (β = 0.74, *P* < 0.01) and anxiety symptoms (β = 0.65, *P* < 0.01) after controlling sociodemographic variables. Moral injury significantly mediated the relationship between spirituality and both depression and anxiety, explaining 60% (0.46/0.76) of the total association between spirituality and depression and 58% (0.38/0.65) of the association with anxiety. No moderating effect of moral injury was found on the spirituality–mental health relationship.

**Conclusions:**

Although they were the findings of a cross-sectional study, these results suggest that concern over transgressing moral values during the pandemic may have been a driving factor for negative mental health symptoms among Chinese health professionals for whom spirituality was somewhat important. Future longitudinal studies are needed to determine the causal nature of these relationships.

## Moral injury and mental health

Moral injury among first-line healthcare workers during the COVID-19 pandemic has received considerable attention during the past year.^1,2^ Litz et al defined moral injury as ‘perpetrating, failing to prevent, bearing witness to, or learning about acts that transgress deeply held moral beliefs’.^3^ Moral injury is thought to be a common experience characterised by negative responses such as shame, guilt, self-condemnation, feelings of betrayal, difficulty trusting and difficulty forgiving.^4^ Previous studies of war veterans have found that moral injury consistently coexists with suicide, depression, post-traumatic stress disorder (PTSD) and substance use disorders.^5–7^ Those who suffer from potentially morally injurious events, such as killing or wounding others, engaging in retribution or disproportionate violence, may have psychological injuries and injuries to their spiritual health and well-being (i.e. the sense of peace and fulfilment in life, and feeling that life has purpose and meaning).

## Healthcare professionals face risk of moral injury

Although the concept of moral injury originally arose in military settings among those suffering from wartime trauma acquired during combat, moral injury may also occur among healthcare professionals, particularly when clinicians feel their ability to deliver care is compromised by the systems being implemented by hospitals, clinics and other organisations (e.g. insurance, reimbursement, electronic health records).^8,9^ Physicians and nurses can be exposed to potentially morally injurious events in which they face ethical dilemmas related to agonising life–death decisions. Physicians have often been asked to decide between life and death by allocating life-saving medical resources between patients in hospitals where medical devices (ventilators), personal protective equipment and lifesaving medications are in short supply, particularly during the COVID-19 pandemic. This may be associated with a sense of helplessness, shame and guilt when hundreds of patients under their care die each day and nothing can be done to save them.^10^ Owing to a recent increase in patient–physician mistrust and violence against physicians,^11^ moral injury may also be a consequence of the violence that physicians experience due to perceived betrayal by the patients they serve. In China, violence against healthcare workers has been recognised as a public health concern owing to the possible triggering factors, which include patients experiencing long waits for short appointments, suspicions that expensive investigations or medications might benefit the doctor or hospital more than themselves, media revelations of poor practice and of questionable relationships between some doctors and industry, and limited means of recourse for dissatisfaction.^12^ Many physicians have been killed or injured during the past decade owing to such violence, beginning even before COVID-19.^13^ At least one qualitative study has suggested that the term ‘moral injury’ is useful for exploring medical students’ experience when participating in their emergency medicine rotations.^14^

## Spirituality, moral injury and mental health

Spirituality is an awareness of the metaphysical, the religious, and has to do with sources of meaning and purpose in life. In practice, spirituality includes but is not limited to participation in organised religion, meditation, prayer, contemplative reflection and activities fostering self-growth, meaning, purpose, and connections with others and with nature.^15^ Moral injury is often associated with spiritual suffering and a need to find hope, trust, reconnection, reconciliation and wholeness.^16^ Spiritual beliefs may provide coping resources (cognitive, social) and rituals (confession, repentance) for dealing with moral transgressions. Studies have found that spirituality is inversely associated with moral injury in US veterans^17^ and US healthcare professionals,^18^ and it may help to moderate the effects of moral injury on PTSD symptoms or promote recovery when such difficulties emerge.^19^ Spiritual beliefs, however, may also contribute to self-condemnation for failure to live up to the high moral standards advocated by religious bodies, thereby contributing to moral injuries.^20^ Several interventions have been developed to address moral injury in the context of PTSD,^21^ including a spiritually-integrated version of cognitive processing therapy that targets moral injury among veterans and active-duty military with PTSD symptoms.^22,23^

To our knowledge, no research has yet examined the relationship between spirituality and moral injury among physicians and nurses in mainland China. The present study seeks to explore the associations among spirituality, moral injury and mental health in a national sample of largely secular Chinese healthcare professionals using an online survey. We hypothesised that: (a) spirituality would be positively associated with moral injury scores; (b) spirituality would be positively related to depressive and anxiety symptoms in this secular sample; and (c) moral injury would help to explain (mediate) the relationship between spirituality and depressive/anxiety symptoms.

## Method

### Participants and procedure

Complete details on enrolment of participants have been described elsewhere.^24^ In brief, a total of 4003 healthcare professionals were recruited from mainland China using a snowball sampling technique. An online survey was conducted between 27 March and 26 April 2020, about 1 month after the COVID-19 pandemic reached its peak in mainland China. Of those approached, 3975 participants agreed to complete the survey; of them, 968 were excluded owing to having been in practice for less than 2 years, having two or more items missing on study measures, or giving the same or similar answer across all items (indicating a lack of thoughtfulness when responding to the questions). As a result, 3006 participants were included in the final analysis.

Inclusion criteria were (a) being a physician or nurse and (b) length of time in practice at least 2 years. Exclusion criteria were (1) history of 6 months or more during the past 2 years of an extended break from practice for any reason; (2) inability to use the internet or other mobile device owing to vision or other physical health problem preventing the completion of an online questionnaire; and (3) not formally licensed to practice medicine or nursing in China.

### Ethics statement

All procedures contributing to this work complied with the ethical standards of national and institutional committees on human experimentation and with the Helsinki Declaration of 1975, as revised in 2008. All procedures involving human subjects were approved by the institutional review board of Ningxia Medical University (protocol #2020-112).

### Consent statement

The survey was designed as anonymous, and participants had to provide online consent before proceeding.

### Measures

Information on sociodemographic characteristics was collected, including age, gender, marital status, education, ethnicity (Chinese Han or minorities), specialty, work area, and length in practice.

Moral injury was assessed using the 10-item Moral Injury Symptom Scale-Health Professional (MISS-HP) developed by Mantri and colleagues (2020).^25^ This measure assesses ten dimensions of moral injury: betrayal, guilt, shame, moral concerns, loss of trust, loss of meaning, difficulty forgiving, self-condemnation, spiritual struggle and loss of faith. Response options for each of the ten items range from 1 to 10, indicating agreement or disagreement, resulting in a total score that ranges from 10 to 100, with higher scores indicating greater moral injury.^26^ The MISS-HP was translated into Chinese following a standard procedure.^27^ Cronbach's alphas in the present sample were 0.71 in nurses and 0.70 in physicians.

The assessment of mental health included measures of depressive and anxiety symptoms using the nine-item Patient Health Questionnaire (PHQ-9) for depression and the seven-item Generalized Anxiety Disorder (GAD-7) scale. Both PHQ-9 and GAD-7 are rated on a four-point Likert scale from 0 (not at all) to 3 (nearly every day).^28,29^ Higher scores indicate more severe symptoms of depression or anxiety. Chinese versions of the PHQ-9 and GAD-7 have been shown to have acceptable internal and test–retest reliability as well as construct and factor analytic validity in medical and general population settings.^30,31^ Cronbach's alphas in the present sample were 0.91 for the PHQ-9 and 0.94 for the GAD-7.

Spirituality was assessed using a visual analogue scale from 0 (not at all important) to 100 (very important) after asking the question: ‘In general, how important are spiritual beliefs in your daily life?’ This question is commonly used to assess spirituality in secular societies and has been strongly associated with psychological well-being.^32^

Religious affiliation was also determined by asking the question: ‘What is your religion?’ Religious affiliation was categorised for analysis into four groups: 1 = none, 2 = Chinese religion (Buddhist, Daoist, etc.), 3 = Western religion (e.g., Christian), and 4 = Muslim.

### Statistical analyses

Descriptive statistics were used to determine average scores with s.d. values and ranges for continuous variables and numbers and percentages for categorical variables. Bivariate analyses were conducted using Pearson's *r* for continuous variables and student's *t*-test or analysis of variance for comparisons of continuous variable scores across categorical variable responses.

Hierarchical linear regression modelling was used to examine the mediating effect of moral injury in the relationship between spirituality and mental health (depression and anxiety), controlling for demographic variables. First, associations between spirituality and mental health (depression and anxiety separately) were determined after controlling for demographic variables (model 1); this was followed by adding moral injury to the model (model 2). Finally, an interaction term between moral injury and spirituality was added to model 2 to determine whether moral injury moderated the relationship between spirituality and mental health states (model 3).

Bootstrap methods of the PROCESS procedure in SPSS designed by Hayes^33^ were employed to examine the mediating effect of moral injury in the relationship between spirituality and depression/anxiety. The IBM SPSS 23.0 version was used to perform all analyses. The alpha level was set to 0.05 for statistical significance.

## Results

As indicated in Table 1, the final sample consisted of 583 nurses and 2423 physicians. Participants were 65% female, 12.3% were minorities, nearly two-thirds (62.5%) provided in-patient care, and most held a bachelor's degree in their field (in China, a bachelor's degree is sufficient to practice medicine). The average age of participants was 35.4 years (s.d. = 8.1), ranging from 20 to 70 years. The average length of practice was 11.6 years (ranging from 2 to 50 years). Most participants (89.2%) indicated no religious affiliation; among those having a religion, Islam was the most common religious affiliation. Spirituality scores ranged from 0 to 100, with an average of 38.5 (s.d. = 28.5) and a median score of 40. Christians reported the highest score on spirituality (72, s.d. = 27), Buddhist/Taoists reported similar scores to those of Muslims (58, s.d. = 27 *v*. 59, s.d. = 25, respectively), and those who indicated no religion reported the lowest scores (35, s.d. = 27).

**Table 1:** Characteristics of sample and bivariate correlates with moral injury in health professionals (*n* = 3006)

Variable	*N* (%)	Mean (s.d.)	MISS-HP,^a^ mean (s.d.)	*Β* (95% CI)^b^
Gender				
Female	1957 (65.1)		46.7 (12.4)	0.72 (−0.27,1.72)
Male	1049 (34.9)		47.4 (13.2)	
Marital status				
Unmarried	656 (21.8)		48.4 (12.1)**	−0.67 (−1.80,0.46)
Married	2266 (75.4)		46.5 (12.8)	
Divorced/widowed	84 (2.8)		46.3 (12.3)	
Ethnicity				
Han	2637 (87.7)		46.9 (12.7)	−0.10 (−1.48,1.27)
Minorities	371 (12.3)		46.9 (12.1)	
Nurse/physician				
Nurse	583 (19.4)		46.2 (12.2)	0.91 (−0.37, 2.18)
Physician	2423 (80.6)		47.1 (12.8)	
Work area				
In-patient	1878 (62.5)		46.9(12.8)	−0.21 (−0.75,0.32)
Out-patient	714 (23.8)		47.0 (12.6)	
ICU/emergency	280 (9.3)		46.8 (12.6)	
Other	134 (4.5)		48.1 (10.8)	
Education				
Bachelor	2029 (67.5)		47.1 (12.5)	0.44 (−0.33,1.22)
Master	813 (27.0)		46.8 (13.0)	
Ph.D.	164 (5.5)		45.8 (13.2)	
Age (range = 20–70), years		35.4 (8.1)	−0.082**	−0.12 (−0.19, −0.06)**
LOP (range = 2–50), years		11.6 (8.5)	−0.085**	−0.11 (−0.17, −0.05)**
Religious affiliation				
No	2680 (89.2)		46.8 (12.7)	0.42 (0.25, −0.30)
Christian/Catholic	29 (1.0)		47.7 (11.2)	
Buddhist/Taoist	103 (3.4)		48.6 (13.5)	
Islam	194 (6.5)		47.5 (11.7)	
Depression (range = 0–30)		10.6 (6.0)	0.437**	0.91 (0.84,0.99)**
Anxiety (range = 0–24)		8.3 (5.3)	0.406**	0.96 (0.88, 1.04)**
Spirituality (range = 0–100)		38.5 (28.5)	0.074**	2.41 (1.65. 3.18)**

MISS-HP, Moral Injury Symptoms Scale-Health Professional; ICU, intensive care unit; LOP, length of practice.

a. Pearson correlation (r) was used to determine the association between MISS-HP and continuous variables; Student's t-test or analysis of variance was used to compare the average MISS-HP scores across categorical variables.

b. General linear model used to control the covariates.

** *P* ≤ 0.01.

Bivariate analyses are presented in Table 1. Unmarried participants scored higher on the MISS-HP compared with those who were married, divorced or widowed (*P* < 0.01). Older participants and those who had spent longer in practice scored lower on moral injury symptoms. Both depressive (PHQ-9) and anxiety (GAD-7) symptoms were strongly and positively related to MISS-HP scores (*P* < 0.01). Spirituality was related to higher MISS-HP scores. No significant difference in moral injury symptoms was found between those having a religious affiliation and those without an affiliation. The associations between spirituality and moral injury persisted in the multivariate analysis after controlling for sociodemographic variables.

As indicated in Table 2, no significant difference was found in PHQ-9 or GAD-7 scores among groups based on religious affiliation. Multivariate analyses revealed that spirituality was positively correlated with depressive symptoms (*β* = 0.74, *P* < 0.01) and with anxiety symptoms (*β* = 0.65, *P* < 0.01). Results of the mediation analysis are presented in Table 3, controlling for age, gender, education, marital status and work area. The mediation effect of moral injury on the relationship between spirituality and both depression and anxiety was significant (*P* < 0.001). Moral injury explained 60% (0.46/0.76) of the total association between spirituality and depression and 58% (0.38/0.65) of the association between spirituality and anxiety. As demonstrated in Table 4, moral injury did not moderate the association between spirituality and either depression or anxiety (interaction for depression, *β* = −0.01, *P* = 0.55; interaction for anxiety, *β* = −0.01, *P* = 0.26) (model 3).

**Table 2:** Correlations of spirituality and mental health conditions in healthcare professionals (*n* = 3006)

Variable	Depression (PHQ)		Anxiety (GAD)	
	Mean (s.d.)/*r*^a^	*Β* (95% CI)^b^	Mean (s.d.)/*r*^a^	*Β* (95% CI)^b^
Religious affiliation				
No	10.5 (6.0)	0.21 (−0.12, 0.55)	8.2 (5.3)	0.08 (−0.21,0.38)
Christian	11.9 (5.4)		8.8 (5.5)	
Buddhist/Taoist	10.9 (6.6)		8.6 (5.7)	
Islam	11.5 (5.6)		8.7 (4.6)	
Spirituality^c^ (range = 0−100)	0.043*	0.74 (0.24, 1.24)**	0.034	0.65 (0.21, 1.09)**

PHQ, Patient Health Questionnaire; GAD, Generalised Anxiety Disorder scale.

a. Pearson correlation (*r*) was used to determine associations between spirituality and continuous variables; student's t-test or analysis of variance was used to compare average PHQ scores and GAD scores across religious affiliation groups.

b. General linear model used to control covariates (gender, marital status, ethnicity, work area, education, length of practice).

c. Logarithm-transformed score to fit normal distribution.

*P < 0.05, **P < 0.01.

**Table 3:** Model examining the mediating effect of moral injury on the relationship between spirituality and depression/anxiety

Effect	*Β*	s.e.	*P*-value	Bias-corrected 95% CI	
				Lower	Upper
Depressive symptoms					
Total effect	0.76	0.18	<0.001	0.42	1.11
Indirect effects	0.46	0.08	<0.001	0.30	0.63
Direct effects	0.30	0.16	0.064	−0.02	0.62
Anxiety symptoms					
Total effect	0.65	0.16	<0.001	0.34	0.95
Indirect effects	0.38	0.07	<0.001	0.24	0.52
Direct effects	0.27	0.14	0.067	−0.02	0.55

**Table 4:** Moderating effect of moral injury on the relationship between spirituality and depression/anxiety

Variables	Model 1		Model 2		Model 3	
	*P*-value	*Β* (95% CI)	*P*-value	*Β* (95% CI)	*P*-value	*Β* (95% CI)
Depressive symptoms						
Constant	<0.001	11.25 (10.0,12.4)	0.020	1.58 (0.25, 2.91)	0.425	0.97 (−1.42, 3.37)
Spirituality^a^	0.001	0.85 (0.33,1.37)	0.180	0.29 (−0.13, 0.73)	0.337	0.73 (−0.76, 2.22)
MISS-HP	–	–	<0.001	0.20 (0.18, 0.22)	<0.001	0.21 (0.17, 0.22)
Spirituality × MISS-HP	–	–	–	–	0.552	−0.01 (−0.04, 0.02)
Model *R*^2^/Δ*R*^2^	–	0.010/–	–	0.193/0.184**	–	0.193/0.000
Anxiety symptoms						
Constant	<0.001	9.89 (9.04,10.75)	0.158	0.85 (−0.33, 2.05)	0.877	−0.17 (−2.32, 1.98)
Spirituality^a^	0.001	0.69(0.26, 1.11)	0.238	0.23 (−0.15, 0.62)	0.075	0.97 (−0.36, 2.23)
MISS-HP	–	–	<0.001	0.16 (0.15, 0.18)	<0.001	0.19 (0.14, 0.23)
Spirituality × MISS-HP	–	–	–	–	0.259	−0.01 (−0.04, −0.01)
Model *R*^2^/Δ*R*^2^	–	0.010/–	–	0.167/0.166**	–	0.168/0.001

Model 1, independent variables include demographical variables; Model 2, Model 1 + MISS-HP; Model 3, model 2 + interaction; MISS-HP, Moral Injury Symptoms Scale-Health Professional.

a. Logarithm-transformed score to fit normal distribution.

** *R*^2^ change test *P* < 0.01.

## Discussion

To our knowledge, this is the first study to explore the relationship between spirituality, moral injury and mental health (depression/anxiety) in a large sample of healthcare professionals during the COVID-19 pandemic in mainland China. We found that spirituality was positively associated with moral injury, depressive symptoms and anxiety symptoms. These findings are not consistent with previous reports indicating that spirituality was inversely associated with moral injury in US veterans and healthcare professionals living in a religious society.^18,19^ These conflicting results may be due to the measure of moral injury used in the present study, which assesses psychological symptoms and struggles with beliefs; these may be more common among those with religious faith, especially in a highly secular country.^23^ Several studies conducted in mainland China have reported a positive relationship between spirituality and mental health problems.^34,35^ Previous studies have also found that Chinese health professionals with a Buddhist/Taoist religious affiliation experienced a higher risk of moral injury than those with no religious preference.^36^ One possible explanation is that having a Buddhist/Taoist religious affiliation may indicate higher moral standards that need to be lived up to, making these health professionals particularly vulnerable to moral injury. Chinese health professionals who are religious may feel abandoned or punished by God when experiencing difficult life situations, resulting in a higher degree of moral distress.^37^ At least one study has reported that religious and moral issues among minority Muslims in Germany generated stress among women, leading to a higher level of mental distress.^38^ More religious healthcare professionals may be driven by higher values and morals that make them feel more responsible to ‘do something’ to help others. When faced with system-related problems that prevent them from doing this, they may feel helpless and therefore develop symptoms of anxiety or depression.

Multivariate analyses in the current study found that moral injury was positively associated with both depression and anxiety symptoms. Moral injury has been consistently shown to coexist with adverse mental health outcomes including suicide, depression, PTSD, and substance misuse in military populations.^39,40^ Although the present study used a cross-sectional design that prevented causal inferences, spiritual-based interventions directed at moral injury have effectively reduced PTSD and other negative emotions among military personnel.^22,23^ Likewise, Mantri and colleagues found in a sample of US healthcare professionals that moral injury was associated with significantly higher depression and anxiety,^18^ as in the present study.

We considered two pathways by which moral injury might affect the relationship between spirituality and depression/anxiety: mediation or moderation. Results from the mediation model indicated that moral injury explained a majority of the variance in the relationship between spirituality and both depression and anxiety. The findings suggest that more spiritual Chinese health professionals may have been more sensitive emotionally to moral concerns over transgressing moral boundaries while carrying out their duties during the COVID-19 pandemic, where decisions might have involved who to treat and who not to treat, given the limited resources available. Finally, we did not find that moral injury moderated the relationship between spirituality and depression/anxiety, indicating that the relationship between spirituality and depression/anxiety was positive regardless of the level of moral injury.

Although religion (as an important component of spirituality) has been shown to increase levels of hope in US veterans and active-duty military,^41^ this dynamic may be difficult to demonstrate in a largely secular population of Chinese healthcare professionals owing to differences in the cultural background. Repeated moral injury experience over time may also cause Chinese healthcare professionals to become more resistant to morally injurious events, as suggested by the finding that healthcare professionals who were older or had been in practice longer reported fewer moral injuries.

The findings here provide preliminary evidence to support addressing spiritual issues when treating Chinese healthcare professionals struggling with moral injury arising during the COVID-19 pandemic.^21^ Doing so may help to improve depressive and anxiety symptoms in these healthcare providers during the pandemic and other major public health events associated with potentially morally challenging decisions, which Chinese healthcare professionals will inevitably face in the future.

### Study limitations

Several limitations affect the interpretation and generalisability of the findings reported here. First, the non-random sampling method used to select participants may affect the ability to generalise results to healthcare professionals across China. Second, the cross-sectional design prevents causal inferences being drawn regarding the relationships among spirituality, moral injury and depression/anxiety found here. Finally, as all measures were self-rated, the accuracy of responses cannot be guaranteed, although previously established reliable and valid measures were used in the assessment of moral injury, depression and anxiety symptoms.

### Implications

Spirituality was positively correlated with moral injury and poor mental health in this sample of Chinese healthcare professionals during the height of the COVID-19 pandemic. Moral injury mediated a significant proportion of the associations between spirituality and both depression and anxiety, suggesting that concern over transgressing moral values during the pandemic may have been a driving force in generating depression and anxiety symptoms among these Chinese healthcare professionals. Although future longitudinal studies are needed to determine the causal nature of these relationships, the present findings provide preliminary evidence that may help to justify the addressing of spiritual issues when treating Chinese healthcare professionals with symptoms of depression and anxiety during the COVID-19 pandemic.

## Data Availability

All data generated or analysed during this study are included in this published article. Additional requests can be made to the corresponding author at wzhzh_lion@126.com.
